# Healthcare Transition From Pediatric to Adult Care Among Youth Living With HIV in South Carolina: Retrospective Study From 2005 to 2021

**DOI:** 10.1016/j.jadohealth.2026.03.002

**Published:** 2026-05-05

**Authors:** Fanghui Shi, Huiyi Xia, Katherine E. Weaver, Xiaoming Li, Bankole Olatosi, Sharon Weissman, Rebecca Widener, Xueying Yang

**Affiliations:** a South Carolina SmartState Center for Healthcare Quality, Arnold School of Public Health, University of South Carolina, Columbia, South Carolina; b Department of Health Promotion, Education and Behavior, Arnold School of Public Health, University of South Carolina, Columbia, South Carolina; c Department of Psychology, University of South Carolina College of Arts and Sciences, Columbia, South Carolina; d Department of Health Services Policy and Management, Arnold School of Public Health, University of South Carolina, Columbia, South Carolina; e Department of Internal Medicine, School of Medicine, University of South Carolina, Columbia, South Carolina; f Department of Pediatrics, School of Medicine, University of South Carolina, Columbia, South Carolina

**Keywords:** Youth with HIV, Healthcare transition, Electronic health records, Successful transition, Perinatally acquired HIV, Nonperinatally acquired HIV

## Abstract

**Purpose:**

As more youth with HIV (YWH) survive into adulthood, successful healthcare transition (HCT) from pediatric to adult care is crucial for optimal health outcomes. This study aimed to characterize YWH transition status and identify individual- and county-level predictors.

**Methods:**

Statewide HIV surveillance data in South Carolina were used for analysis. The first outcome, “ever transition,” indicated whether a patient had at least one adult HIV care visit after the last pediatric care before age 26. Among those who transitioned, a successful transition was defined as timely linkage and retention in care. Logistic regression models identified individual and county-level predictors of HCT outcomes.

**Results:**

Among 658 YWH, 538 (89.8%) transitioned, with the mean (standard deviation) age at diagnosis being 17.3 (6.02) years and age at first adult transition being 21.8 (2.02) years. Among 493 YWH who ever transitioned, 215 (43.6%) transitioned successfully. YWH ever transitioned were more likely to be diagnosed at an older age (adjusted odds ratio [aOR] = 1.187, 95% confidence interval [CI]: 1.066–1.332) and without a history of psychiatric diagnoses (aOR = .276, 95% CI: .104–790). Youth with perinatally acquired HIV were more likely to transition successfully than those with nonperinatally acquired HIV (aOR = 4.124, 95% CI: 1.689–10.548), and individuals in counties with higher social vulnerability in socioeconomic status had lower odds of successful transitions (aOR = .693, 95% CI: .491–.968).

**Discussion:**

To improve successful HCT, tailored transition plans are needed for youth with nonperinatally acquired HIV and living in economically disadvantaged areas.

HIV diagnoses among youth aged 13–24 in the United States have more than doubled in the last 15 years, with youth accounting for 19% of new HIV diagnoses in 2022. [1,2] As more youth with HIV (YWH) survive into adulthood due to advances in antiretroviral therapy (ART), healthcare transition (HCT) from pediatric care to adult care has emerged as a critical public health issue with implications for ongoing efforts to end the HIV epidemic in the United States. [3–5] HCT marks the beginning of a new era in care when they may have a new provider and are expected to demonstrate more initiative and self-management. [6–8] A successful HCT is essential to maintain continuous engagement in care, improve the youth’s ability to independently manage their disease, and ultimately decrease HIV-related morbidity and mortality rates. [9,10].

Despite the importance of HCT, there is no consensus on the definition of a successful HCT for YWH. Providers generally agree that successful HCT is multifaceted, involving both behavioral and serologic indicators. [11] However, the conceptualization of “success” in research has varied considerably. For example, one study in South Africa found that 23% of YWH had successful HCT, which was defined as being retained in care and virally suppressed 1 year after HCT. In contrast, a US-based study reported a 37% success rate using the criterion of attending a single adult care visit within 9 months of HCT. [12] One study proposed that “success” in HCT is characterized as attending two adult medical appointments and having two measures of viral indicators. [8] Additionally, some literature classified HCT outcomes simply as “successful” or “failed” without a consistent end date. [10,12,13].

YWH face numerous barriers throughout the HCT process at both individual and structural levels. [14,15] For example, individually, lack of health insurance poses a major obstacle, which can be exacerbated if insurance coverage changes at the time of transition. [8,16] Unlike many European countries with universal health coverage, many adolescents in the United States are at risk for losing insurance when they reach 18 years of age under public programs or 26 years old if they are covered under a parent’s private health insurance plan. In a study of perinatally acquired young adults’ post-transition, half of the participants who had self-discontinued ART cited insurance as the primary reason. [17] Furthermore, YWH who are marginalized and stigmatized due to their sexual orientation, gender identity, or racial/ethnic minority status may be at risk for psychosocial complications, which subsequently affect the HCT success. [18,19] At the structural level, clinicians have frequently cited the limited accessibility of adult clinics as a barrier to HCT. [20] Furthermore, YWH who live in rural areas or low-income neighborhoods may face additional barriers to a successful HCT. [16,20] Specifically, in the southern United States, YWH may experience multiple structural vulnerabilities (e.g., poverty, residential segregation, unequal access to HIV treatment services, and restricted Medicaid expansion), which might have been exacerbated during the COVID-19 pandemic. [21] Therefore, monitoring the implementation and progress of the transition process is essential to improve the successful HCT and to contribute to efforts to end the HIV epidemic.

HIV disproportionately burdens the Southern states in the United States due to their higher rates of poverty, structural barriers to healthcare, and healthcare provider shortage. [21–23] Previous research has investigated HCT in YWH in Southern states but has either focused on YWH with one transition status, such as linkage to care, as the transition endpoint, or a narrow scope of predictors at the individual level. [24] Other studies have explored transition readiness but failed to characterize participants’ subsequent transition patterns. [25] Finally, some research examined contextual factors associated with HCT, but they were either qualitative or did not quantify the transition status. [26] These literature gaps call for research that examines YWH at a population level and explores the use of a system-level approach to explore factors related to HCT status beyond the individual level. There is also a need to examine care outcomes beyond the initial visit with an adult provider. Using integrated statewide population-based datasets, this study aimed to evaluate HCT outcomes in YWH in South Carolina (SC) and examine the association of individual- and structural-level characteristics with HCT outcomes.

## Methods

### Data sources and linkage

We used the SC Enhanced HIV/AIDS Reporting System (eHARS), a statewide population-based HIV surveillance system, to identify all YWH in SC from January 1st, 2005, to June 30th, 2021, and obtain their history of HIV acquisition, treatment, and laboratory results over time. To ensure sufficient follow-up, we restricted the cohort to individuals diagnosed before June 2019, allowing for at least 2 years of postdiagnosis observation for each participant. SC eHARS is a laboratory-based reporting system to which all statewide Cluster of Differentiation 4 (CD4) count and viral load (VL) tests have been mandatorily reported since January 1, 2004. [27,28] The identifiable link from eHARS was sent to the SC Revenue and Fiscal Affairs Office (RFA), which served as the honest broker for the linkage of all diagnoses in the Uniform Billing form (UB-92) format from all emergency departments, hospital inpatient facilities, ambulatory-care facilities, and outpatient surgery facilities in SC.

For data security, the RFA used deidentified system-generated ID numbers to ensure confidentiality but allowed the study to conduct data mining at both the individual and aggregated levels. To obtain regional population characteristics (e.g., percentage of Black individuals), the deidentified dataset from RFA was linked to publicly available datasets (i.e., the American Community Survey and the Centers for Disease Control and Prevention [CDC]’s Agency for Toxic Substances and Disease Registry) using the Federal Information Processing Standards code, a unique identifier for each county. This study was approved by the Institutional Review Boards at the University of SC and relevant SC state agencies (Pro00131130), which granted a waiver of informed consent due to the use of deidentified secondary data.

### Study cohort

**Figure 1.**
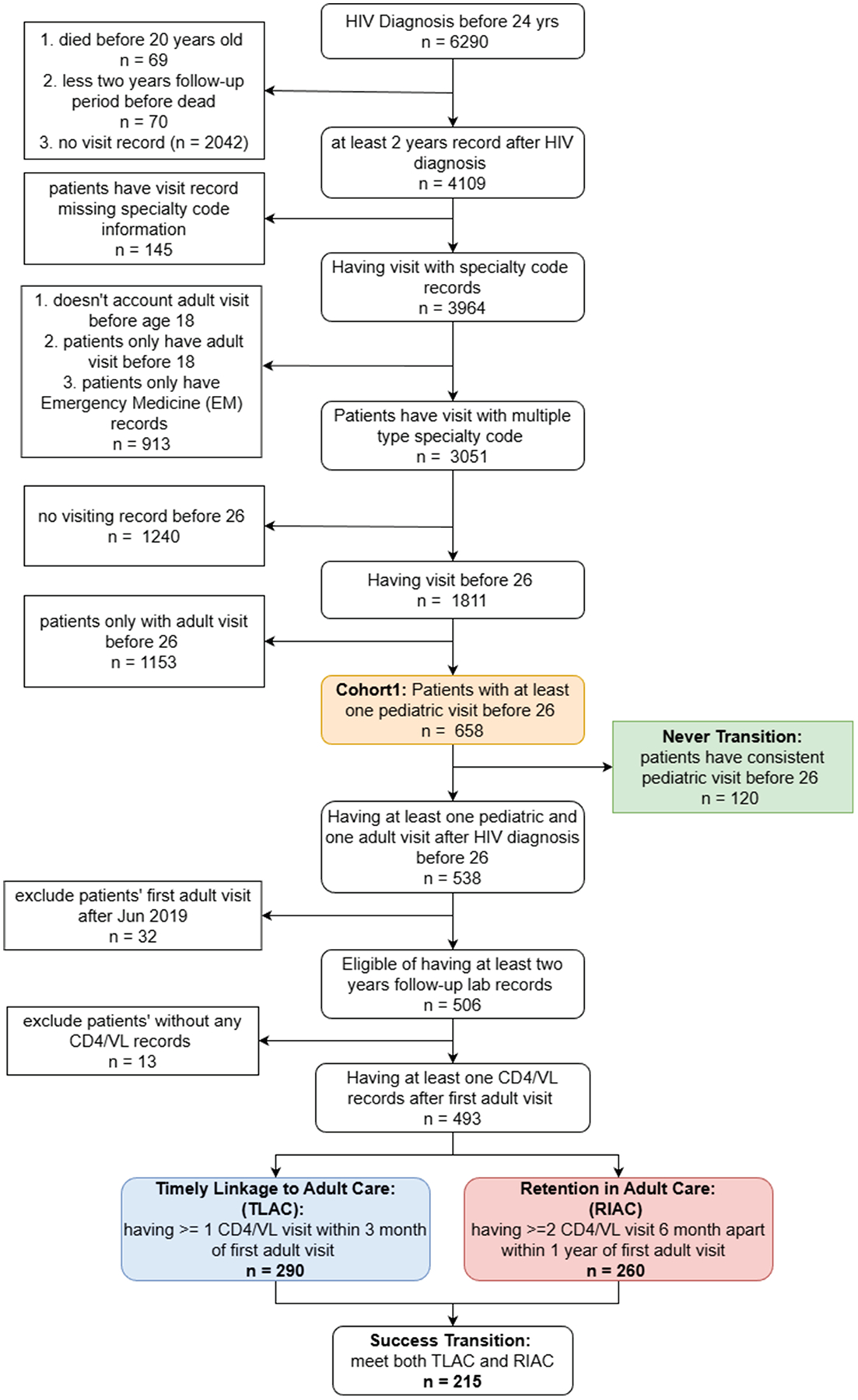
The flowchart of cohort construction.

For the first study outcome, ever transition, we examined all youth PWH who (1) were diagnosed with HIV before age 24 years old in SC; (2) survived until 20 years old; (3) had at least 2 years follow-up time after diagnosis; and (4) had at least one pediatric visit after HIV diagnosis and before 26 years. For the second study outcome, successful transition, we conducted analyses among YWH who transitioned from pediatric to adult care before age 26 and with at least one CD4 count or viral load record after the first adult visit. To determine if and when a patient made a transition from pediatric to adult care, we categorized facilities into two categories: pediatric and adult care. Facilities were classified as pediatric HIV care sites if they were known by HIV surveillance staff to provide pediatric care, or if their names included terms such as “pediatric,” “children,” or “adolescent.” Then, a clinician was counselled to determine the remaining facilities based on the facility names. Specifically, visit records with specialty codes unrelated to HIV (i.e., obstetrics & gynecology, obstetrics, maternal & fetal medicine, otolaryngology, and general surgery) before 19 years old were not considered as adult care. Additionally, patients with only emergency medicine records were excluded from the study cohort. The process of cohort identification was illustrated in Figure 1.

### Study outcomes of interest

Our first study outcome is “ever transition,” a binary outcome indicating whether a patient had at least one adult HIV care after the last pediatric visit and before age 26 (0 = never transitioned, 1 = transitioned). Among those who transitioned and had at least one CD4 count or viral load record after the first adult visit, the second study outcome is “successful transition,” a binary outcome (0 = no, 1 = yes) indicating whether an individual met two post-transition criteria, including timely linkage to care and retention in care. Timely linkage to care was defined as having at least one adult clinical visit or viral load/CD4 count measure within 3 months of their last pediatric care. Retention in care required ≥2 consecutive adult clinic visits or viral load/CD4 count measures 6 months apart within 1 year after transition. [29] Individuals who were both timely linked to care and retained in care were considered to have a successful transition.

### Covariates

We examined individual-level covariates from three categories, including: (1) demographic characteristics: age at HIV diagnosis (years old), age at HCT (years old), sex (male and female), and race/ethnicity (non-Hispanic Black, non-Hispanic White, and Hispanic/unknown); (2) HIV-related characteristics: CD4 count (<200 cell/mm^3^, 200–500 cell/mm^3^, and ≥500 cell/mm^3^), HIV viral load (≤200 copies/ml and >200 copies/ml), and likely HIV acquisition category (heterosexual, men who have sex with men/injected drug use, perinatal exposure, and others). Specifically, for individuals who never transitioned, we extracted the information about CD4 count and viral load before the last pediatric visit prior to age 26. For individuals who transitioned, we extracted information about CD4 count and viral load before the first adult care after the pediatric care; and (3) other health conditions: historical comorbidities (measured by the Charlson comorbidity index score and categorized into <2 and ≥2) and psychiatric diagnosis (yes and no). [30,31] The psychiatric diagnosis was defined as a binary variable indicating whether an individual had at least one of the following mental health disorders: bipolar disorder, depression, schizophrenia, anxiety, dementia, schizoaffective disorders, persistent mood disorder, obsessive-compulsive disorder, and personality disorder. [31] Both historical comorbidities and psychiatric diagnoses were defined based on corresponding International Classification of Diseases-9/10 codes [31].

For the second study outcome, we additionally obtained six county-level variables: three social vulnerability subindices (i.e., socioeconomic status, minority status and language, and housing type and transportation) and three healthcare resources-related characteristics (i.e., the percent of healthcare employment, shortage of primary care physicians, and shortage of mental healthcare providers). Specifically, social vulnerability in socioeconomic status was calculated based on the proportions of people below the poverty line, income, unemployment, and lack of a high school diploma. The index of minority status and language was based on the proportion of racial/ethnic minority residents and English proficiency. The index of housing type and transportation was based on proportions of persons in multiunit structures, crowding, mobile homes, institutionalized group quarters, and no household vehicle access. [32] All three social vulnerability subindices ranged from 0 to 1, with higher scores representing greater vulnerability. [32] As for the value of Health Professional Shortage Area code related to shortage of primary care physicians and mental health care providers, “0” indicated “none of the county designated as a shortage area,” “1” indicated “whole county designated as a shortage area,” and “2” means “one or more parts of county designated as shortage area.”

### Statistical analysis

First, we assessed the socio-demographic distribution (e.g., count number and percentage for categorical characteristics and mean and standard deviation [SD] for continuous characteristics) by ever transition status and by successful transition status. Categorical characteristics were compared using χ^2^ and Fisher’s exact tests, and continuous variables were compared using an independent samples *t*-test or Mann—Whitney test as appropriate. Logistic regression models were employed to explore the relationship between individual-level characteristics and ever transition status. County-level variables were not included in this model because most YWH in the study were diagnosed before 2009, when county-level characteristics were not available. For successful transition status, we conducted logistic regression models to examine both individual- and county-level factors. We performed all analyses in R (RStudio, Boston, MA), and a *p* value < .05 was considered statistically significant.

## Results

### Demographic distributions by ever transition and successful transition status

A total of 658 YWH were included in this study, and 100 (15.2%) of them were perinatally acquired. A total of 538 (81.8%) YWH transitioned from pediatric care to adult care, with the mean (SD) age at diagnosis being 17.3 (6.02) years and the mean age at transition being 21.8 (2.02) years. Among YWH who transitioned, most of them were male (67.8%), non-Hispanic Black (84.6%), and had no psychiatric diagnosis (94.2%). There is no significant difference in historical comorbidities, CD4 count, and viral load between YWH who never transitioned and those who transitioned (*p* value larger than 0.05 for all) (Table 1).

Among 493 YWH who ever transitioned and with at least one viral load or CD4 count record after the first adult care, 215 (43.6%) transitioned successfully. The demographic distribution in YWH with and without successful transition was similar (*p* values larger than 0.05), with most of them being male (65.6% vs. 68.0%), non-Hispanic Black (83.7% vs. 85.3%), and with no psychiatric diagnosis (89.3% vs. 92.8%). The mean age at transition for individuals who transitioned successfully (21.6 [SD = 2.19]) and unsuccessfully (22.0 [SD = 2.19]) was also not significantly different (*p* value = .051). Regarding county-level characteristics, the mean value of social vulnerability in socioeconomic status was smaller in YWH with successful transition (0.577 [SD = 0.231]) than those without successful transition (0.616 [SD = 0.243]) (Table 2).

### Factors associated with ever transition and successful transition

Compared to YWH who never transitioned from pediatric care to adult care before age 26, those who transitioned were more likely to be older at diagnosis (adjusted odds ratio [aOR] = 1.187, 95% confidence interval [CI]: 1.066–1.332) and have no psychiatric diagnosis (aOR = 0.276, 95% CI: .104–.790) (Table 3). Among YWH who transitioned, those with unsuppressed viral load (aOR = 0.559, 95% CI: 0.342–0.908) and living in counties with higher social vulnerability in socioeconomic status (aOR = 0.693, 95% CI: 0.491–0.968) were less likely to transition successfully. Compared to YWH who acquired HIV through heterosexual contact, YWH who were perinatally acquired were more likely to transition successfully (aOR = 4.124, 95% CI: 1.689–10.548) (Table 4).

## Discussion

To our knowledge, this study is the first to use statewide population-based large-scale data to assess HCT status among YWH in SC over an extended period, and to examine both individual- and county-level characteristics related to HCT outcomes. We found that approximately 81.8% of YWH transitioned from pediatric care to adult care, and among those who transitioned, only 43.6% achieved a successful transition. In comparison, the percentage of successful transitions reported varied widely in other US-based studies, ranging from 37% to 60%. [10,13] This variation is largely attributable to differences in how successful transition is defined across studies, as well as differences in the study population. Younger age at HIV diagnosis and a history of psychiatric diagnoses were identified as potential barriers to ever transitioning. Regarding successful transition, compared to youth with nonperinatally acquired HIV, those with perinatally acquired HIV were more likely to transition successfully. Additionally, residing in counties with higher social vulnerability in socioeconomic status emerged as a structural barrier. Identification of these risk factors or barriers could help policymakers and service providers improve systems and services to better support YWH during HCT and ultimately decrease HIV-related morbidity and mortality rates among them.

In this study, both age at HIV diagnosis and history of psychiatric diagnoses emerged as significant individual-level predictors of ever transitioning to adult HIV care before age 26. The prolonged engagement in pediatric care in individuals diagnosed at a younger age may delay the recognition or prioritization of the HCT planning. To improve timely transition and engagement of care, it is important to develop early and developmentally appropriate HCT strategies, such as structured transition readiness assessments and proactive communication about adult care expectations, particularly for YWH diagnosed at earlier ages. [33] Additionally, YWH may face an increased burden of mental health disorders, which substantially affect the HCT success. [18] A systematic review of the literature on mental health among adolescents with HIV found that psychiatric disorders such as anxiety and depression are more prevalent among adolescents with HIV than among adolescents without HIV. [34] Most pediatric healthcare providers in the United States expressed concern that YWH who had mental health problems would be at risk of being lost in care in the process of transition, especially if the adult care services do not have the same multidisciplinary and integrated care models as the pediatric care services. [18] Additionally, when transitioning into adult care, YWH may anticipate additional combined mental health challenges, such as feelings of loss due to separating from the pediatric provider, anxiety about increasing autonomy, and difficulty navigating the adult healthcare system, which can further impede the transition process. [35] Structured and supportive transition processes, such as formalized practices or rituals to acknowledge and support this transition, may help address emotional support needs during this period. [36].

This study found that youth with nonperinatally acquired HIV (e.g., through heterosexual contact) were less likely to transition successfully than those with perinatally acquired HIV, which was consistent with most previous studies. [37,38] While both groups may face similar challenges (e.g., fear of HIV disclosure), youth with nonperinatally acquired HIV may encounter additional stressors. These include concerns about stigma or discrimination associated with engaging in risk exposures (e.g., unprotected sex). [39] Furthermore, youth with newly diagnosed HIV are often identified at a later stage and may present with opportunistic and/or sexually transmitted infections, further complicating the transition process. Compared with youth with nonperinatally acquired HIV, youth with perinatally acquired HIV who disclosed their HIV status earlier may be more likely to access necessary social support, fostering a sense of belonging and improving engagement in care. [40] In addition, they may be more accustomed to the clinic care environment and have intense preparations for the transition into adult care. [41] Given the diverse needs of HCT for youth with nonperinatally and perinatally acquired HIV, more specific guidelines that distinguish them and address their differences are needed.

In examining factors associated with successful transition, our study found that YWH residing in counties characterized by higher social vulnerability in socioeconomic status were less likely to achieve a successful transition. A lack of economic resources in economically disadvantaged areas, such as SC, can impact prioritizing competing needs like food and housing over adherence to care among YWH, and may also affect access to essential healthcare components needed for the transition process, such as insurance coverage and availability of adult care providers. [13,42,43] Thus, beyond improving individual-level readiness for transition, it is critical to incorporate geographically tailored interventions that address contextual socioeconomic disadvantages that may limit successful transition. [11,35].

This study is subject to several limitations. First, major mental illnesses, such as bipolar disorder and schizophrenia, may have distinct impacts on HCT outcomes compared with more prevalent mental health conditions, such as anxiety and depression. However, the small number of participants with bipolar disorder or schizophrenia in this study precluded examination of condition-specific differences. Second, some critical factors―such as food insecurity, knowledge and attitude about transition, and psychosocial support―were not controlled in the multivariable modeling due to data unavailability. Further studies are needed to better understand how these factors impact HCT to inform effective interventions and better support YWH as they undergo transition. Third, our approach allowed us to differentiate healthcare visits into pediatric and adult care, but it cannot distinguish whether the services were HIV-related or other types of healthcare. To mitigate this limitation, we excluded some visit records with specialty codes unrelated to HIV care (i.e., obstetrics & gynecology, obstetrics, maternal & fetal medicine). Fourth, youth mobility data were not available in our dataset. Thus, we could not capture the mobility of young people who may leave the state for educational or economic opportunities. Therefore, some HCTs may have occurred outside the state, and the findings in the current study should be interpreted with caution.

### Conclusions

This study highlighted individual (i.e., age at HIV diagnosis and HIV risk categories) and county-level (i.e., socioeconomic vulnerability) factors associated with HCT status in SC. Consideration of these factors is critical in future intervention, clinical practice, and policy research that seek to understand barriers to HCT from multiple levels. To promote timely and successful HCT, tailored transition plans spanning the pre-transition (pediatric care), transition, and post-transition (adult care) periods are needed for YWH who were diagnosed at a younger age, have a history of mental health conditions, acquired HIV non-perinatally, and reside in economically disadvantaged areas.

## Figures and Tables

**Table 1:** Demographic distribution by ever transtions status

Characteristics	Ever transition (N = 658)		
	No (n = 120)	Yes (n = 538)	*p* value
Age at HIV diagnosis (years)			
Mean (SD)	12.8 (9.16)	17.3 (6.02)	<.001
Age at transition (years)			
Mean (SD)		21.8 (2.02)	
Sex			
Female	37 (30.8%)	173 (32.2%)	.863
Male	83 (69.2%)	365 (67.8%)	
Race			
Non-Hispanic White	15 (12.5%)	61 (11.3%)	.001
Non-Hispanic Black	90 (75.0%)	455 (84.6%)	
Hispanic/Asian/unknown	15 (12.5%)	22 (4.1%)	
HIV risk category			
Heterosexual	-	100 (18.6%)	<.001
MSM/IDU	58 (48.3%)	324 (60.2%)	
Perinatal exposure	41 (34.2%)	59 (11.0%)	
Others (a)	-	55 (10.2%)	
Charlson comorbidity index			
<2	-	483 (89.8%)	.647
≥ 2	-	55 (10.2%)	
CD4 count (cell/mm3)			
>500	47 (39.2%)	186 (34.6%)	.098
200–500	-	184 (34.2%)	
<200	-	61 (11.3%)	
Missing	36 (30.0%)	107 (19.9%)	
Viral load level (copies/mL)			
<200	48 (40.0%)	198 (36.8%)	.2
≥200	40 (33.3%)	229 (42.6%)	
Missing	32 (26.7%)	111 (20.6%)	
Psychiatric diagnosis			
No	-	507 (94.2%)	.398
Yes	-	31 (5.8%)	

(a) Combined category including (1) adult no identified risk, (2) adult no risk reported, (3) child no identified risk, (4) child rcvd clotting factor, and (5) child other confirmed risk.

Counts less than 10 cannot be displayed due to data dissemination policy and some additional data were obscured to prevent secondary calculation of these values.

MSM = men who have sex with men; IDU = injected drug use.

**Table 2:** Demographic distribution by success transition status

Characteristics	Successful transition (N = 493)		
	No (n = 278)	Yes (n = 215)	*p* value
Age at transition (years)			
Mean (SD)	22.0 (1.92)	21.6 (2.19)	.051
Sex			
Female	89 (32.0%)	74 (34.4%)	.641
Male	189 (68.0%)	141 (65.6%)	
Race			
Non-Hispanic White	30 (10.8%)	25 (11.6%)	.884
Non-Hispanic Black	237 (85.3%)	180 (83.7%)	
Hispanic/Asian/unknown	11 (4.0%)	10 (4.7%)	
HIV risk category			
Heterosexual	62 (22.3%)	33 (15.3%)	<.001
MSM/IDU	174 (62.6%)	120 (55.8%)	
Perinatal exposure	16 (5.8%)	35 (16.3%)	
Others (a)	26 (9.4%)	27 (12.6%)	
Comorbidity conditions			
< 2	231 (83.1%)	176 (81.9%)	.812
≥ 2	47 (16.9%)	39 (18.1%)	
CD4 count (cell/mm3)			
>500	100 (36.0%)	67 (31.2%)	.02
200–500	96 (34.5%)	77 (35.8%)	
<200	22 (7.9%)	35 (16.3%)	
Missing	60 (21.6%)	36 (16.7%)	
Viral load level (copies/mL)			
<200	90 (32.4%)	87 (40.5%)	.13
≥200	128 (46.0%)	89 (41.4%)	
Missing	60 (21.6%)	39 (18.1%)	
Mental health condition			
No	258 (92.8%)	192 (89.3%)	.228
Yes	20 (7.2%)	23 (10.7%)	
SVI-socioeconomic			
Mean (SD)	0.616 (0.243)	0.577 (0.231)	.067
SVI-minority/language			
Mean (SD)	0.762 (0.086)	0.768 (0.091)	.444
SVI-housing type/transportation			
Mean (SD)	0.769 (0.149)	0.762 (0.154)	.641
Shortage of mental healthcare providers			
Not shortage	10 (3.6%)	-	.813
Whole county shortage	77 (27.7%)	-	
Partial shortage	191 (68.7%)	153 (71.2%)	
Shortage of primary care physicians			
Whole county shortage	66 (23.7%)	41 (19.1%)	.255
Partial shortage	212 (76.3%)	174 (80.9%)	
Percent of healthcare employment			
Mean (SD)	0.146 (0.040)	0.150 (0.039)	.243

(a) Combined category including (1) adult no identified risk, (2) adult no risk reported, (3) child no identified risk, (4) child rcvd clotting factor, (5) child other confirmed risk.

Counts less than 10 cannot be displayed due to data dissemination policy, and some additional data were obscured to prevent secondary calculation of these values.

MSM = men who have sex with men; IDU = Injected drug use; SVI = Sociovulnerability index.

**Table 3:** The association of individual-level characteristics with ever transition status

Characteristics	Ever transition (yes vs. no)	
	Crude OR (95% CI) (a)	Adjusted OR (95% CI) (b)
Age at diagnosis (years)	1.082 (1.055, 1.110)***	1.187 (1.066, 1.332)**
Sex		
Female	Ref	Ref
Male	0.941 (0.608, 1.434)	0.568 (0.254, 1.252)
Race		
Non-Hispanic White	Ref	Ref
Non-Hispanic Black	1.243 (0.655, 2.235)	1.05 (0.408, 2.462)
Hispanic/Asian/unknown	0.361 (0.150, 0.858)*	0.19 (0.053, 0.646)**
HIV risk category		
Heterosexual	Ref	Ref
MSM/IDU	0.503 (0.225, 1.005)	0.476 (0.109, 1.661)
Perinatal exposures	0.13 (0.055, 0.275)***	1.225 (0.136, 11.027)
Others	0.413 (0.159, 1.037)	0.514 (0.105, 2.354)
Comorbidity conditions		
0/1	Ref	Ref
≥ 2	1.253 (0.644, 2.683)	1.025 (0.392, 3.064)
CD4 count (cell/mm3)		
>500	Ref	Ref
200–500	1.661 (1.001, 2.797)	1.368 (0.736, 2.571)
<200	1.713 (0.825, 3.927)	1.835 (0.693, 5.310)
Viral load level (copies/mL)		
≤ 200	Ref	Ref
>200	1.388 (0.876, 2.210)	0.989 (0.526, 1.86)
Mental health condition		
No	Ref	Ref
Yes	0.673 (0.331, 1.484)	0.276 (0.104, 0.79)*

(a) Crude OR was estimated from separate unadjusted models for each variable.

(b) Adjusted OR was estimated from a multivariable model including all covariates simultaneously.

OR = odds ratio; CI = confidence interval.

* *p*-value <0.05;

** *p*-value <0.01;

*** *p*-value <0.001.

**Table 4:** The association of individual- and county-level characteristics with successful transition status

Characteristics	Successful transition (yes vs. no)	
	Crude OR (95% CI) (a)	Adjusted OR (95%CI) (b)
Age at transition (years)	0.915 (0.837, 0.999)*	0.888 (0.783, 1.006)
Sex		
Female	Ref	Ref
Male	0.897 (0.615, 1.311)	1.018 (0.408, 2.554)
Race		
Non-Hispanic White	Ref	Ref
Non-Hispanic Black	0.911 (0.518, 1.617)	1.542 (0.732, 3.348)
Hispanic/Asian/unknown	1.091 (0.392, 3.014)	2.462 (0.664, 9.695)
HIV risk category		
Heterosexual	Ref	Ref
MSM/IDU	1.296 (0.803, 2.119)	1.456 (0.515, 4.152)
Perinatal exposure	4.11 (2.011, 8.704)***	4.124 (1.689, 10.548)*
Others	1.951 (0.983, 3.903)	1.882 (0.757, 4.704)
Residence		
Urban	Ref	Ref
Rural	1.550 (0.65,4.252)	1.087 (0.466,2.542)
Comorbidity conditions		
0/1	Ref	Ref
≥ 2	1.089 (0.679, 1.738)	0.931 (0.505, 1.703)
CD4 count (cell/mm3)		
>500	Ref	Ref
200–500	1.197 (0.777, 1.847)	1.295 (0.794, 2.120)
<200	2.374 (1.288, 4.459)**	3.415 (1.650, 7.227)**
Viral load level (copies/mL)		
≤ 200	Ref	Ref
>200	0.719 (0.481, 1.074)	0.559 (0.342, 0.908)*
Mental health condition		
No	Ref	Ref
Yes	1.545 (0.824, 2.922)	0.891 (0.394, 1.976)
SVI-socioeconomics	0.853 (0.712, 1.020)	0.693 (0.491, 0.968)*
SVI-minority/language	1.073 (0.898, 1.288)	0.853 (0.636, 1.140)
SVI-housing type/transportation	0.958 (0.802, 1.146)	1.283 (0.959, 1.734)
Shortage of mental healthcare providers		
Not shortage	Ref	Ref
Whole county shortage	0.877 (0.324, 2.441)	1.188 (0.273, 5.243)
Partial shortage	1.001 (0.385, 2.690)	0.951 (0.257, 3.566)
Shortage of primary care physicians		
Whole county shortage	Ref	Ref
Partial shortage	1.321 (0.855, 2.061)	1.679 (0.953, 3.012)
Percent of healthcare employment	1.113 (0.930, 1.337)	0.985 (0.760, 1.277)

OR = odds ratio; CI = confidence interval; SVI = Sociovulnerability index.

(a) Crude OR was estimated from separate unadjusted models for each variable.

(b) Adjusted OR was estimated from a multivariable model including all covariates simultaneously.

* *p*-value <0.05;

** *p*-value <0.01;

*** *p*-value <0.001.

## Data Availability

The authors are prohibited from making individual-level data available publicly due to provisions in our data use agreements with state agencies/data providers, institutional policy, and ethical requirements. To facilitate research, we make access to such data available via approved data access requests through the data owners. The data are unavailable externally or for re-release due to prohibitions in data use agreements with our state agencies or other data providers. Institutional policies stipulate that all external requests for data access require collaboration with a USC researcher. For more information or to make a request, please contact Bankole Olatosi, PhD: Olatosi@mailbox.sc.edu. The underlying analytical codes are available from the authors upon request.
